# Experimental-Theoretical Investigation of the Strength and Deformability of Full-Scale Second-Generation Profiled Sheeting Samples

**DOI:** 10.3390/ma18184365

**Published:** 2025-09-18

**Authors:** Volodymyr Semko, Nataliia Mahas, Pavlo Semko, Serhii Skliarenko, Roman Rabenseifer

**Affiliations:** 1 Department of Structural Engineering, Institute of Building Engineering, Faculty of Civil and Transport Engineering, Poznan University of Technology, Piotrowo Street 5, 61-138 Poznan, Poland; 2 Department of Building Construction, Faculty of Civil Engineering, Slovak University of Technology in Bratislava, Radlinského 2766/11, 810 05 Bratislava, Slovakia; nataliia.mahas@stuba.sk (N.M.); roman.rabenseifer@stuba.sk (R.R.); 3 Department of Building and Civil Engineering, Educational and Research Institute of Architecture, Civil Engineering and Land Management, National University «Yuri Kondratyuk Poltava Polytechnic», Vitaliya Hrytsayenka Street, 24, 36011 Poltava, Ukraine; 4 Department of Computer Technologies in Construction, Faculty of Architecture, Construction and Design, State University «Kyiv Aviation Institute», Liubomyra Huzara 1, 03058 Kyiv, Ukraine; serhii.skliarenko@npp.kai.edu.ua

**Keywords:** cold-formed steel structures, steel trapezoidal profiled sheeting, full-scale test, deflection, EN1993-1-3

## Abstract

Steel trapezoidal profiled sheeting (STPS) is widely used in the construction industry. It is often viewed not as a structural element but as a material with its own properties, similar to bricks or masonry blocks. Consequently, to determine the load-bearing capacity and deformability of STPS, engineers most often rely on tables developed at the request of manufacturers. In Europe, these tables are compiled according to the EN 1993-1-3 methodology. However, there is a notable lack of studies comparing the results of theoretical calculations and manufacturer-provided data with the outcomes of experimental tests on full-scale profiled sheeting samples. For this reason, the authors conducted a study of 12 full-scale specimens of second-generation trapezoidal sheeting. The samples were tested under a two-span scheme, with deflections measured at the midpoint of each span and settlement of the sheeting at the supports. The study found that half the specimens showed higher deformability in the elastic stage than predicted, with differences of 16% to 105% based on span length and sheet thickness. For 11 out of 12 tested specimens, the onset of plastic deformations occurred before the samples reached their theoretical load-bearing capacity. The main novelty is identifying differences between Eurocode calculations and experimental results, showing higher deflections and earlier plastic deformations in tests. These full-scale STPS results offer both scientific and practical value.

## 1. Introduction

Steel trapezoidal profiled sheeting (STPS) is widely used in construction worldwide due to its simple manufacturing, transportation, and installation. Quite often, designers consider STPS not as a structure consisting of individual structural elements (flat plates, intermediate stiffeners of various shapes, and corner elements), but as a material with specified characteristics. The main design characteristics for profiled sheeting are the load-bearing capacity of 1 m^2^ of sheeting for a given span and the load-bearing capacity of 1 m^2^ of STPS for a given span and deflection values. The design of profiled sheeting in most European countries is carried out in accordance with the requirements of EN 1993-1-3 [1,2]. Sheeting is divided into three generations, differing in the presence of longitudinal and transverse stiffeners [3]. First-generation STPS uses only flat plates. Second-generation adds longitudinal stiffeners in flanges or webs, affecting stress–strain and requiring adjusted design methods.

**Figure 1:**
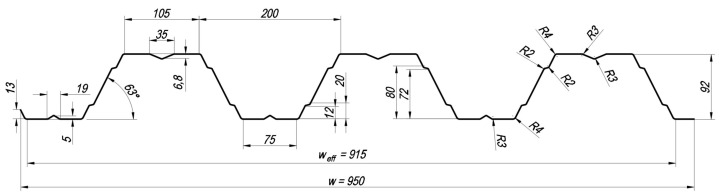
Cross-sectional drawing of the experimental specimens, all dimensions in mm.

In most cases, profiled sheets are used as horizontal load-bearing elements in single-, two-, or three-span schemes. In the transverse direction, the connection of the sheeting occurs by overlapping the edge of the sheet without a stiffener (left edge in Figure 1) onto the edge of the adjacent sheet, which has a stiffener (right edge in Figure 1). It creates a parameter known as the effective width of the sheeting (in the figure, w_eff_ = 915 mm), which characterises the efficiency of material use in the cross-section [4]. Other methods of connecting sheeting along the longitudinal edges are also known [5]. It is possible to increase the length of the load-bearing structure by combining several sheets into a multi-span continuous structure. The multi-span nature of the sheeting is achieved by overlapping the sheets over the support and fastening the webs of the sheets using fastening elements. Overlapping the sheeting not only allows for the formation of a structure with a large number of spans but also strengthens the sheeting at the support, increasing resistance to the combined action of bending moment and support reaction. Studies on this type of strengthening are dedicated to the works of Biegus & Czepiżak [6,7]. Well-known works are devoted to investigating the influence of moment and concentrated force on the resistance of trapezoidal sheeting [8,9,10,11].

STPS are quite often used as components of composite structures, such as steel-concrete and facade or roofing sandwich panels. The works [12,13,14,15] present the results of studies on the performance of STPS within steel-concrete composite structures. The works [16,17] present the results of tests on sandwich panels subjected to explosion loads, utilising both numerical and experimental methods.

It should be noted that published studies almost entirely lack results of tests on full-scale STPS under conditions close to operational scenarios. In most tests, short fragments are used, with loads applied through a system of traverses, as seen in the works of Biegus & Czepiżak [7], Budescu, Ciongradi & Roşca [18], and Casafont, Marimon, Bové, Ferrer & Centelles [19]. The use of short specimens allows for reduced research costs and testing time. However, it primarily enables the investigation of the resistance of the studied sheeting type to effects of interest to researchers.

Among the results of tests on full-scale specimens, notable findings include those from Rzeszut & Szumigała [20,21]. The authors investigated the strength and deformability of single-span sheeting samples with wave heights of 135 and 153 mm and a span of 5900 mm. In [20], the graphs in Figure 3 [20] show that the actual deflection values exceed those declared by the sheeting manufacturer. It should be emphasised that the conducted investigations covered only a narrow group of sheeting—single-span members with a fixed span length and constant support width. Moreover, the obtained results do not include a comparison with theoretical calculations performed in accordance with the Eurocode provisions. The work [22] presents the results of laboratory tests on BTR 160.250.750 profiled sheeting samples with a thickness of 0.75 mm. A three-span design scheme was used, with a span length of 4 m and support widths of 150 mm. Four specimens made from a single sheet and one reinforced specimen with additional overlays at intermediate supports were tested. The authors concluded that the increase in load-bearing capacity due to reinforcement at the support was 78%. The work [23] presents the results of tests on three-span T153-119L-840 sheeting samples with thicknesses of 0.75 and 0.88 mm, formed by overlapping at intermediate supports. A notable aspect is that four samples were tested, each consisting of two sheets in width, with a span of 6 m.

The relatively limited number of test results for full-scale specimens is sometimes explained by researchers’ obligations to commercial clients not to disclose the obtained results. Many authors from the aforementioned works note discrepancies between the measured vertical deflections, for some specimens, and the results obtained through theoretical methods according to Eurocode 3.

Summarising the review of previous studies, their limitations can be identified. In particular, calibrating design methods on short specimens does not allow for a transfer of the results to theories based on beam models. The behaviour of single-span specimens differs significantly from that of two-span specimens: in single-span specimens, there is a lack of hogging bending moment, no combined action of the bending moment and concentrated force at the middle support, and the influence of the presence and location of longitudinal stiffeners in the webs is different. Thus, the investigation of full-scale STPS specimens opens up an almost unexplored experimental area in the field of cold-formed steel structures.

The aim of this article is therefore to present new experimental results of tests on full-scale two-span specimens, to analyse them and to compare with theoretical calculations according to EN 1993-1-3:2024, as well as to identify specific features in the structural behaviour of two-span steel trapezoidal sheeting.

## 2. Materials and Methods

The research can conditionally be divided into two parts: experimental and theoretical. The experimental part involved testing full-scale specimens on stands constructed directly at the sheeting manufacturing site. The theoretical part of the study involved determining the load-bearing capacity and deformability of the profiled sheets using the Eurocode 3 methodology.

### 2.1. Description of the Experimental Stand, Sheeting Specimens, and Testing Procedure

The primary objective of the tests was to establish the ultimate load-bearing capacity of the profiled sheets on behalf of the manufacturing companies. At the same time, the authors of this work aimed to gather as much data as possible by conducting tests outside laboratory conditions and using a limited set of measuring instruments.

The tested specimens were sourced from two different manufacturers, featuring an identical cross-sectional type (see Figure 1). This paper describes the results of tests on 12 specimens (see Table 1). All presented specimens were tested in the “positive” position, meaning the wider flanges were located at the upper part of the cross-section. The tests were conducted using a two-span design scheme with a uniformly distributed load applied along the span length and sheet width.

The load was applied using artificial weights, specifically silicate or ceramic bricks. This method of load application was chosen because the tests were conducted not in a laboratory, but on a specially constructed test rig located directly at the premises of the sheeting manufacturer. Prior to testing, 100 randomly selected bricks were weighed. For this sample, the average weight of a single brick was determined, which was then used to calculate the mean load value applied to the specimens. The bricks were placed on the sheeting through wooden spacers. The number of load application lines for specimens S01–S03 was 20, for specimens S04–S06 it was 26, for specimens S07–S09 it was 29, for specimens S10 and S11 it was 30, and for specimen S12 it was 32. Since the number of load application lines exceeded 8 (or 4 per span), this method of load application can be considered to simulate a uniformly distributed load.

**Figure 2:**
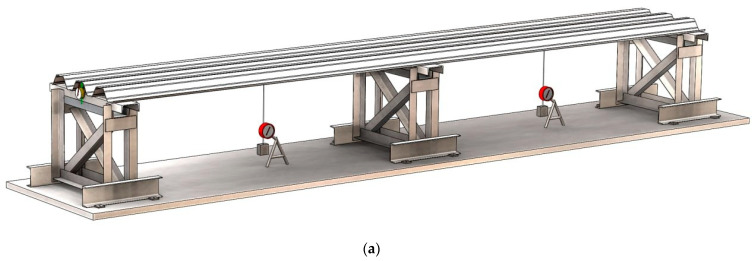

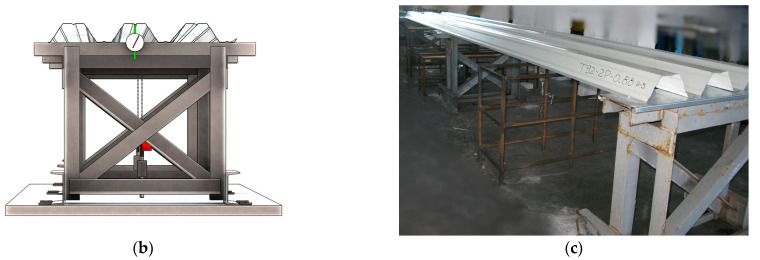
Schematic of the test stand and its general view with the placed specimen: (**a**) General view of the stand; (**b**) Side view of the stand; (**c**) Photo of the stand with specimen S03.

The profiled sheeting specimens represented continuous elements resting on steel supports. The supports were made from hot-formed steel profiles (see Figure 2). The width of the upper support element was 160 mm. By welding a channel section 12 or attaching square tubes 20 × 20 mm on the sides, it was possible to adjust the width of the supports on which the profiled sheeting rested. The supports were installed in a single row and anchored to the concrete floor of the building. Deviations from the horizontal plane of the upper edges of the supports did not exceed 2–3 mm. The height of the steel supports was 800 mm, allowing physical access to install measuring instruments under the specimen, facilitating the placement of weights on the specimen, and enabling the recording of instrument readings.

**Figure 3:**
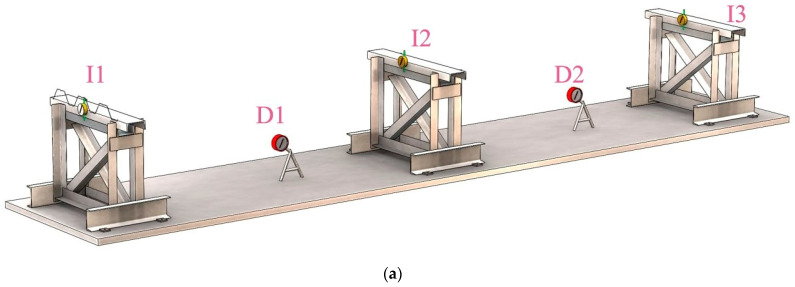

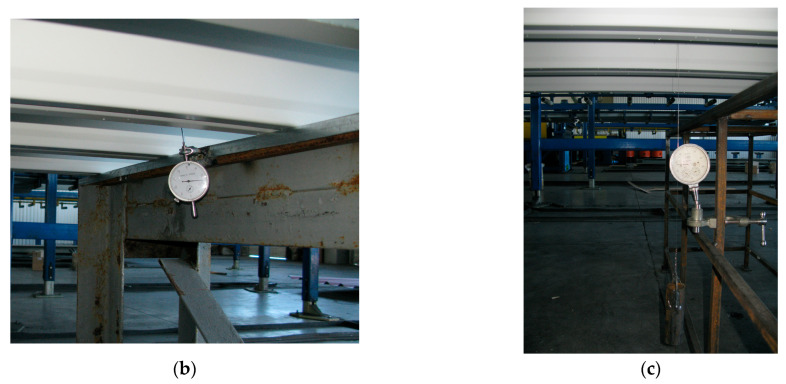
Scheme of the placement of measuring instruments: (**a**) General view of the layout of indicators and deflectometers; (**b**) Photo of an installed indicator on the intermediate support; (**c**) Photo of an installed deflectometer.

The specimen was placed on pre-installed supports and secured to them using self-drilling screws. The self-drilling screws were fastened into each lower flange of the sheeting at the end and intermediate supports. After securing the specimens, the measuring instruments were installed: two “6ПAO” clock-type deflectometers and three “ИЧ-10” clock-type indicators, both with a measurement accuracy of 0.01 mm. The layout scheme of the measuring instruments is shown in Figure 3a. The clock-type indicators (Figure 3b) were installed in the following sequence: indicator I1 on the end support before deflectometer D1 (Figure 3c), indicator I2 on the intermediate support, and indicator I3 on the end support after deflectometer D2. For specimens S07–S09, indicators were not installed. The indicators were directly attached to the steel supports. For securing the deflectometers, additional steel structures were installed, which also served an additional function—acting as supplementary supports in the middle of the spans to ensure safety during testing in case of sudden specimen failure. Before starting the tests, the operation of the measuring instruments was checked, and initial readings were recorded.

**Figure 4:**
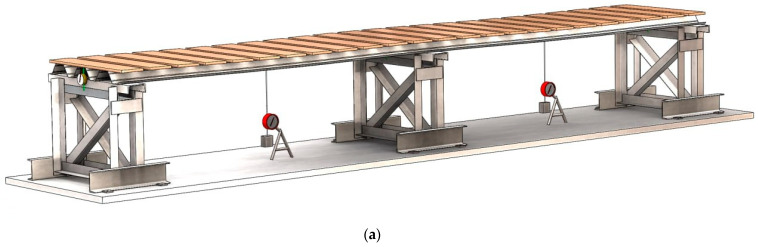

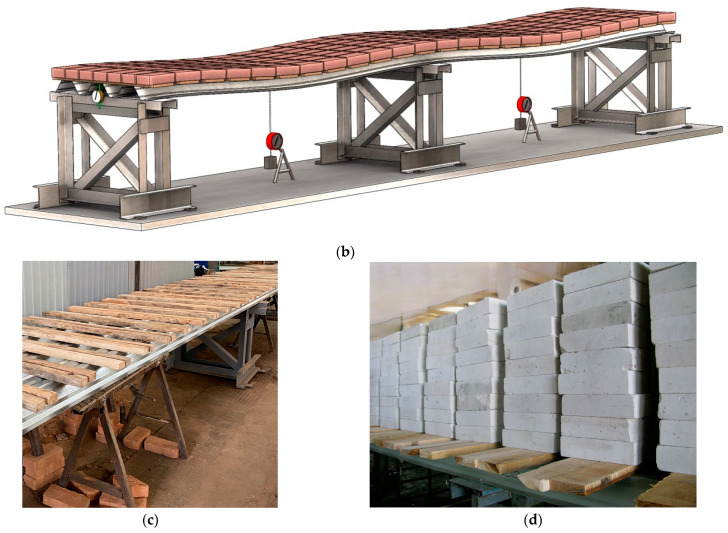
Sequence of specimen loading: (**a**) Placement of wooden spacers on the surface of the profiled sheet; (**b**) Row-by-row placement of bricks; (**c**) Photo of installed wooden spacers for specimen S07; (**d**) Photo of brick rows during loading for specimen S11.

The next step involved placing boards at designated locations, onto which bricks were subsequently laid, and the readings of the measuring instruments were recorded (Figure 4a,c). Following this stage, the loading process began with the placement of weights (bricks). One loading step typically corresponded to one row of bricks (Figure 4b). After placing each row of bricks, readings were taken from the indicators and deflectometers. After recording the readings, the loading procedure was repeated (Figure 4d). The loading continued until the specimen lost its strength or the stability of its individual elements. The failure criterion was the rapid development of plastic deformations at the supports and along the spans, accompanied by the loss of stability of the flat areas of the sheeting cross-section at the intermediate support. In some specimens, progressive failure was observed within the span, particularly in the zone of the maximum bending moment. At the point of failure, the number of placed bricks was recorded. After unloading the specimens, they were inspected, and the nature of the failure was documented.

### 2.2. Theoretical Calculations of Strength and Deformability of Profiled Sheeting Specimens

For the theoretical determination of the strength of profiled sheeting specimens and the assessment of their deformability, the following assumptions were used: (1) the sheeting specimens were modelled as beams supported on three points, with the right-end and middle supports being pinned and restrained against movement, while the left-end support was pinned and allowed to move; (2) the width of the beam was equal to the effective width of the sheet, w_eff_ (see Figure 1); (3) the span lengths were taken as the distances between the midlines of the supports; (4) the values of internal forces (bending moment, shear force) and support reactions were determined using well-known formulas of structural mechanics for two-span beams; (5) the strength of the cross-sections was calculated according to the methods described in EN 1993-1-3:2024 [1]; (6) the maximum deflection of the sheeting was determined based on the effective moment of inertia of the cross-section of the steel profiled sheeting.

The determination of the theoretical strength of the sheeting was carried out according to the methodology of EN 1993-1-3:2024 [1]. In this work, the authors define theoretical strength as the maximum value of uniformly distributed load per 1 m of specimen length, q_max_, which was determined based on the following conditions:

(1) $$q_{max}=min\left\{\begin{array}{c} 8V_{b}/3L;\\ 8R_{w,end}/3L;\\ 4R_{w,mid}/5L;\\ 128M_{p}/9L^{2};\\ 8M_{n}/L^{2};\\ \frac{1}{\frac{L^{2}}{10M_{n}}+\frac{L}{R_{w,mid}}} \end{array}\right\}\;\left[kN/m\right],$$

where V_b_—shear resistance of the cross-section of STPS according clause 8.1.5 [1], kN; R_w,end_—resistance to transverse force at the end of the specimen (edge support) according clause 8.1.6 [1], kN; R_w,mid_—resistance to transverse force at the middle support of the specimen according clause 8.1.6 [1], kN; M_p_—bending resistance of cross-section for the “positive” position according clause 8.1.4 [1], kNm; M_n_—bending resistance of cross-section for the “negative” position according clause 8.1.4 [1], kNm; L—the value of the span, m.

The maximum deflection f_max_ due to the uniformly distributed load q_max_ was determined using the formula

(2) $$f_{max}=\frac{q_{max}L^{4}}{185EI_{eff}},$$

where E—Young’s modulus, I_eff_—moment of inertia of the effective cross-section of the profiled sheeting in the “positive” position.

**Figure 5:**
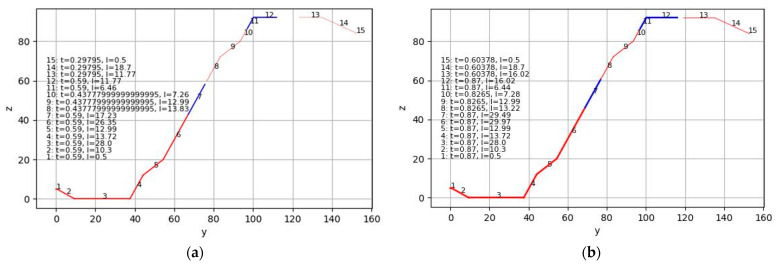
Visualisation of the effective cross-section for one half of the profiled sheeting rib under the action of a bending moment: (**a**) For specimens S01, S04; (**b**) For specimens S07–S09. The letters “y” and “z”, along with adjacent numbers, denote the horizontal and vertical coordinates of the cross-section points, respectively; the letters “t” and “l” denote the thickness and length of the sections, respectively. All dimensions in mm. Red colour indicates the tensioned areas of the cross-section, blue indicates the compressed areas, and thin red lines show the compressed areas of the sheeting that form intermediate stiffening elements in the compressed part of the cross-section.

The effective cross-sectional properties were determined for the geometry presented in Figure 1. The width of the cross-section was taken as w_eff_. The thickness of the steel core of the sheets from which the specimens were made is presented in Table 1. The yield strength value for steel S320GD was adopted according to [1], namely 320 N/mm^2^. The yield strength value for steel DX51 was adopted based on test protocols provided by the manufacturer, namely 293 N/mm^2^. For the calculation of the effective cross-sectional properties and the strength of the profiled sheeting, a programme was written in the Python 3 programming language. In addition to normative literature, the authors used manuals [24,25,26,27,28] for preparing the code. The calculation results are presented in Table 2 and Table 3, and the effective cross-sections for the case of bending about the horizontal axis are shown in Figure 5. It is worth noting that the cross-sections presented in Figure 5 were used to determine the bending resistance in the “positive” position. This case is representative of cross-sections located between the supports. For verifying the bending strength at the middle support, the effective cross-section for the sheet in the “negative” position—where the narrower flanges are in the compressed zone—was used. For determining the effective moment of inertia, cross-sections similar to those shown in Figure 5 were used, but with the thickness of all sections equal to the thickness of the steel core of the sheet “t”. Additionally, to simplify the presentation of results, the theoretical deflection was calculated using the minimum value of the effective moment of inertia, as at low loads on the profiled sheeting, the effective moment of inertia will equal the moment of inertia of the gross cross-section. In this case, the deflection curve will exhibit a bilinear shape.

## 3. Results

**Figure 6:**
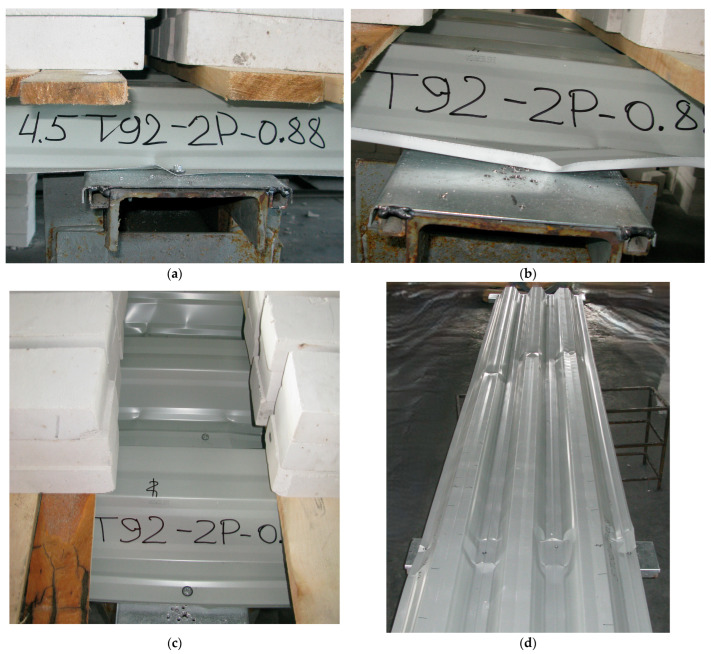
Characteristic deformations of specimens: (**a**) Crumpling of the compressed unstiffened flange at the intermediate support; (**b**) Crumpling of the compressed flange with a stiffening element at the intermediate support; (**c**) Indentation of the bottom flanges of the middle corrugations and deformations of the webs and intermediate stiffening elements at the intermediate support; (**d**) Appearance of half of specimen S12 after testing.

**Figure 7:**
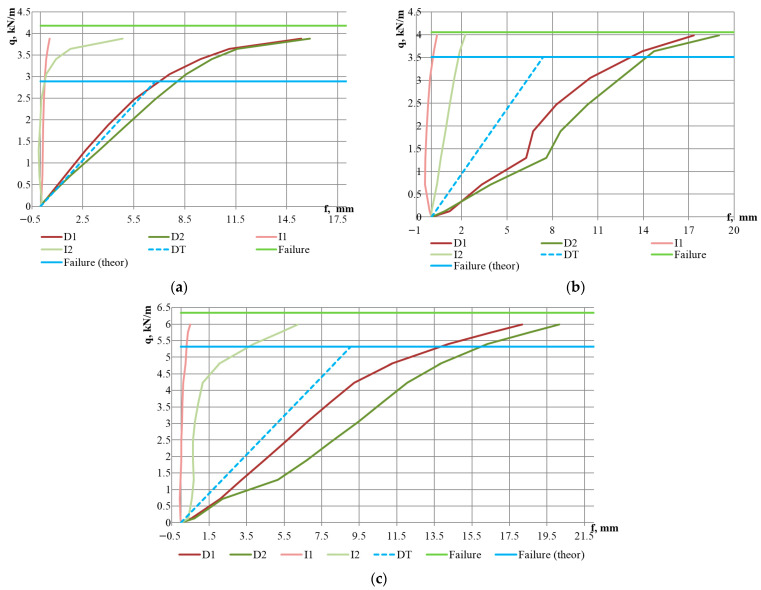
Load–deflection curves of mid-span vertical deflections and reductions in sheeting depth at the supports for specimens with a span of 3 m: (**a**) T92-2P-0.63 (S01); (**b**) T92-2P-0.7 (S02); (**c**) T92-2P-0.88 (S03). Legend: D1, D2—deflectometer readings; I1, I2—indicator readings. The positions of deflectometers and indicators are shown in Figure 3. DT—theoretical deflection values. Failure—experimentally determined failure load. Failure (theor)—theoretically calculated failure load.

**Figure 8:**
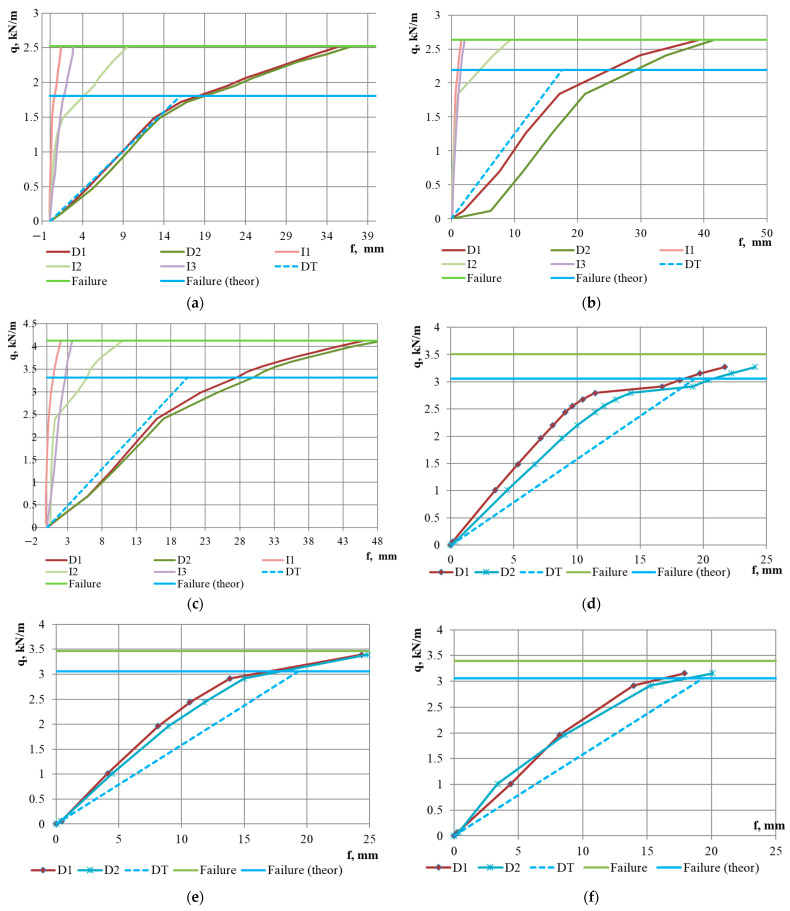
Load–deflection curves of mid-span vertical deflections and reductions in sheeting depth at the supports for specimens with a span of 4 m: (**a**) 4T92-2P-0.63 (S04); (**b**) 4T92-2P-0.7 (S05); (**c**) 4T92-2P-0.88 (S06); (**d**) 4H-92-2P-0.88-1 (S07); (**e**) 4H-92-2P-0.88-2 (S08); (**f**) 4H-92-2P-0.88-3 (S09). Legend: D1, D2—deflectometer readings; I1–I3—indicator readings. The positions of deflectometers and indicators are shown in Figure 3. DT—theoretical deflection values. Failure—experimentally determined failure load. Failure (theor)—theoretically calculated failure load.

**Figure 9:**
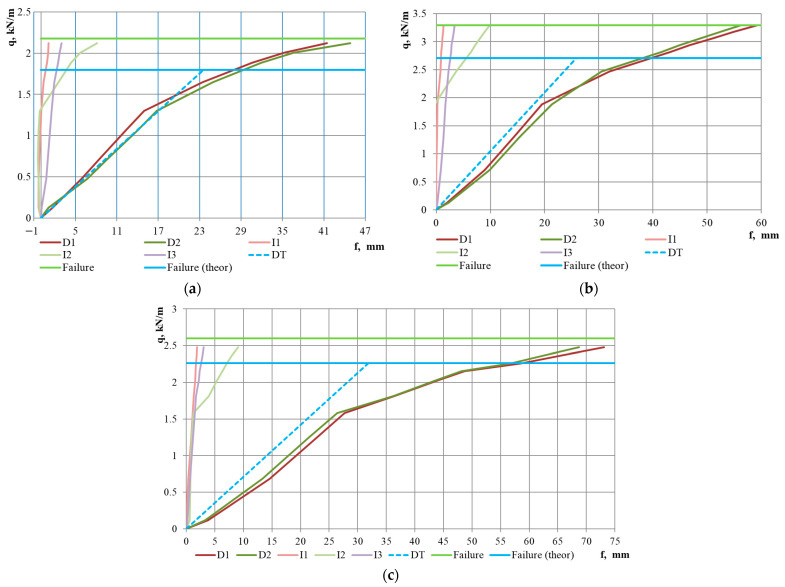
Load–deflection curves of mid-span vertical deflections and reductions in sheeting depth at the supports for specimens with a span of 4.5 m: (**a**) 4.5T92-2R-0.7 (S10); (**b**) 4.5T92-2R-0.88 (S11); and with a span of 5 m: (**c**) 5T92-2P-0.88 (S12). Legend: D1, D2—deflectometer readings; I1–I3—indicator readings. The positions of deflectometers and indicators are shown in Figure 3. DT—theoretical deflection values. Failure—experimentally determined failure load. Failure (theor)—theoretically calculated failure load.

The tests were conducted until the failure of all specimens (Figure 6d). In the initial stages of loading, a linear behaviour was observed (up to a loading level of 59–76% of the ultimate load), as evidenced by the deflection graphs in Figure 7, Figure 8 and Figure 9. At the same time, upward bending of the upper flange of the middle corrugation of the sheeting over the intermediate and/or middle support was noted, as indicated by the negative readings of indicators I1–I3 during the initial loading stages. At this stage, crumpling of the lower flanges of the sheet in the area of the middle support (Figure 6a,b) was also observed, along with local indentation of the lower flanges of the sheeting over the intermediate support. As the load increased, the development of instability in the intermediate stiffening element of the web, located in the lower (compressed) part of the cross-section, was observed (Figure 6c). Concurrently, vertical deformation of the inclined walls of the sheeting due to the support reaction was noted. In the subsequent loading stages, the deflection values increased in a nonlinear relationship with the loads (Figure 7, Figure 8 and Figure 9). Failure of the specimens occurred due to the loss of stability of the flat sections of the sheeting cross-section at the intermediate support. As a result of subsequent force redistribution, progressive failure of specimens S01, S02, S04, S10, and S12 (Figure 6d) occurred in the middle of one of the spans, while for specimen S05, failure was observed in both spans.

The experimental results are presented graphically in Figure 7, Figure 8 and Figure 9. The values of the ultimate loads for the specimens are shown by the light green solid horizontal line (“Failure” line). The dashed line DT indicates the vector from the origin to the value of the maximum theoretical deflection fmax, calculated according to Equation (2) and given in Table 3. A comparative analysis of the theoretical and experimental results is presented in Section 4, “Discussion” of this paper.

It should be noted that a stepped or irregular character of the dial gauge readings at the initial loading stages was observed for specimens S02, S03, and S05. It may be explained by imperfections of the profiled sheeting in the support region or by the quality of the seating of the sheet on the support. For the other specimens, the variation in the deflection readings on both dial gauges was similar, and the deviations between the graphs were insignificant. An interesting observation is that at the moment when the slope of the mid-span deflection curve changes, a significant increase in the incremental vertical deformations recorded by the indicator at the intermediate support (I2) is observed; however, the magnitude of these deformations does not reach the increment of the vertical deformations in the span, which may indicate plastic behaviour of the steel sheeting.

Another noteworthy fact is that the plastic behaviour, which, as mentioned earlier, begins at a load level of 59–76% of the ultimate load (Table 4), accounts for approximately 50% of the total vertical deformation for specimens with a 3 m span and up to 65% for the specimen with a 5 m span.

Regarding the influence of steel grade, it can be noted that for specimens made of DX51 steel, the onset of plastic resistance was observed at a higher load level—80–86% of the ultimate load (Figure 8d–f and Table 4)—and the failure occurred more abruptly. For example, for specimen S09 (Figure 8f), failure took place at the next loading stage immediately following the stage at which the beginning of plastic behaviour was recorded.

## 4. Discussion

Conducting a comparative analysis of the vertical deflection graphs for the specimens—both theoretical and experimental—allows for the conditional identification of three distinct groups of results. The first group of graphs, presented in Figure 7a, Figure 8a and Figure 9a, describes the behaviour of specimens S01, S04, and S10. For this group, a good correlation is observed between the vertical deflection values measured during testing and the theoretical deflection graph. It should be noted that these specimens have the thinnest steel sheet thicknesses, corresponding to spans of 3, 4, and 4.5 m, respectively. The second group of graphs, shown in Figure 8d–f, describes the behaviour of specimens S07–S09, which are made from steel DX51, distinct from the other specimens. The data from these graphs indicate that the experimental vertical displacement values are lower than the theoretical ones. The third group of graphs, not mentioned above, indicates higher experimentally obtained deflection values compared to those calculated theoretically. This group encompasses half of all tested specimens. For the specimens in this group, the difference between the averaged experimental and theoretical deflection values at a load level of 50% of the ultimate load is as follows: 105% for specimen S02, 64% for S03, 45% for S05, 16% for S06, 17% for S11, and 30% for S12. At the point of reaching a load level equal to the theoretical load-bearing capacity of the sheeting, this difference increases to 100% for specimen S02, 62% for S03, 53% for S05, 40% for S06, 58% for S11, and 78% for S12. It confirms the findings of previously mentioned researchers [6,18,20], who noted discrepancies between the magnitude of vertical deflections and results obtained through theoretical calculations according to Eurocode 3. At the same time, these researchers did not pursue further investigation of this issue, as they also had examples of good correlation, such as the results of the first group of graphs in our analysis. In our opinion, the study of the deformability of STPS requires greater attention from researchers. It would be appropriate to conduct an analysis of experimental data for other sheeting cross-sections, and in the event of confirming the poor predictability of vertical deflections, to plan numerical and laboratory experiments to investigate factors that may dangerously affect the deformability of the sheeting. When planning future research, it is worth noting that in a real structure, the profiled steel sheet operates as part of a diaphragm, and its behaviour differs significantly from that of a single sheet [29].

As mentioned earlier, sheeting made from thinner sheets (core thickness of 0.59 mm) demonstrated good agreement between theoretical and experimental deflections. Additionally, for these specimens (S01, S04), the highest correlation was observed between the load at which the onset of plastic deformations (q_pl_) occurred and the theoretical load-bearing capacity (q_theor_) (Table 4), with ratio coefficients q_pl_/q_theor_ of 1.059 and 0.953, respectively. Specimens made from DX51 steel also exhibited high q_pl_/q_theor_ ratio coefficients, ranging from 0.914 to 0.953. For the remaining specimens (7 in total), this ratio varied between 0.723 and 0.87, indicating that the onset of plastic deformations began 13–27% below the theoretical load-bearing capacity, which contradicts the provisions of Eurocode 3 for Class 4 cross-sections, where the operation of structures in the plastic stage is considered as failure (loss of strength).

The smallest difference between the theoretical load-bearing capacity of the sheeting and the experimental data is 9.8–12.8% for specimens S07–S09. For the remaining specimens, this difference ranges from 13% to 30.8%, with the most significant discrepancy observed for specimens S01 and S04, at 30.8% and 28.5%, respectively. This fact, combined with the previously noted good correlation between the loads at which plastic deformation begins and the theoretical capacity of these specimens, suggests a higher reliability of theoretical results specifically for specimens with the thinnest steel sheet thickness.

Analysis of the available literature shows that, to date, there are practically no studies presenting test results of STPS with different cross-section types and configurations. The given sample of 12 two-span specimens is an attempt to demonstrate the relevance of research in this area. Based solely on the existing test results, it is not easy to draw definitive conclusions regarding the parameters of sheeting for which the Eurocode methodology provides comparable results. It is therefore necessary to expand the experimental sample to include sheeting with other cross-section types and structural schemes (single- and three-span), as well as to analyse experimental results obtained by other researchers.

## 5. Conclusions

This work presents the results of an experimental-theoretical study on the strength and deformability of full-scale two-span specimens of second-generation steel trapezoidal profiled sheeting. Accordingly, the results are applicable only to the cases considered in this study. To extend these findings to other structural schemes (single-, three-, or multi-span) and cross-section types, additional research is required. At the same time, the obtained results allow for a preliminary conclusion regarding the potential of experimental studies on full-scale STPS specimens.

It was established that the deformation behaviour of the steel specimens varies depending on the thickness and steel grade. For specimens with the thinnest steel sheet thickness (0.59 mm) made from S320GD steel, the best correlation was observed between the actual vertical deflection values and their theoretical values in the elastic stage. Specimens made from DX51 steel demonstrated lower deformability in the elastic stage than predicted by theoretical calculations. At the same time, half of the tested specimens exhibited greater actual deformability in the elastic stage than the predicted theoretical deflections, with differences ranging from 16% to 105%, depending on the span length and steel sheet thickness of the specimen.

For 11 out of 12 tested specimens, the development of plastic deformations began before the specimens reached their theoretical load-bearing capacity, with the most considerable difference being 27%. It is the most significant finding, as it may indicate an overestimated level of reliability for this type of structure when evaluated according to the requirements of Eurocode 3.

The difference between the theoretical and experimental strength of the profiled sheeting specimens ranges from 9.8% to 30.8%. The smallest difference is characteristic of specimens made from DX51 steel, while the largest is observed for specimens made from S320GD steel with the thinnest sheet thickness.

Based on the theoretical and experimental investigations of second-generation two-span sheeting conducted by the authors, the following recommendations can be formulated for practising engineers: (1) Eurocode may underestimate deflections; (2) Plastic deformations may start at ~80% of theoretical loads.

In future work, the authors plan to analyse experimental and theoretical results for single- and three-span specimens, as well as for other types of cross-sections.

## Figures and Tables

**Table 1:** List of specimens and their main characteristics.

Specimen	Mark of the Specimen	Span, mm	Core Thickness t, mm	Width of the Support (Edge/Intermediate), mm	Steel Grade
S01	T92-2P-0.63	3000	0.59	150/200	S320GD
S02	T92-2P-0.7	3000	0.66	150/200	S320GD
S03	T92-2P-0.88	3000	0.84	150/200	S320GD
S04	4T92-2P-0.63	4000	0.59	150/200	S320GD
S05	4T92-2P-0.7	4000	0.66	150/200	S320GD
S06	4T92-2P-0.88	4000	0.84	150/200	S320GD
S07	4H-92-2P-0.88-1	4000	0.87	120/120	DX51
S08	4H-92-2P-0.88-2	4000	0.87	120/120	DX51
S09	4H-92-2P-0.88-3	4000	0.87	120/120	DX51
S10	4.5T92-2P-0.7	4500	0.66	150/200	S320GD
S11	4.5T92-2P-0.88	4500	0.84	150/200	S320GD
S12	5T92-2P-0.88	5000	0.84	150/200	S320GD

**Table 2:** Resistances to shear, bending, and concentrated force action for the investigated cross-section of profiled sheeting of various thicknesses.

t, mm	f_y_, N/mm^2^	V_b_, kN	R_w,end_, kN	R_w,end_, kN	M_p_, kNm	M_n_, kNm	I_eff_, mm^4^
0.59	320	21.54	4.2	21.54	4.53	4.35	864,188
0.66	320	26.65	5.28	26.65	5.61	5.22	992,937
0.84	320	41.72	8.53	41.72	8.11	7.75	1,309,142
0.87	293	34.79	10.55	34.79	8.05	7.58	1,371,806

**Table 3:** Theoretical values of ultimate load and maximum vertical deflection of the sheeting.

Specimen	q_max_, kN/m	f_max_, mm	Specimen	q_max_, kN/m	f_max_, mm
S01	2.88	6.76	S07	3.06	19.33
S02	3.51	7.39	S08	3.06	19.33
S03	5.32	9.05	S09	3.06	19.33
S04	1.81	16.02	S10	1.80	23.54
S05	2.19	17.51	S11	2.71	25.82
S06	3.31	20.4	S12	2.26	31.88

**Table 4:** Load levels at which the onset of plastic behaviour (q_pl_) was observed, theoretical load-bearing capacity (q_theor_), and experimental load-bearing capacity (q_exp_).

Specimen	q_pl_, kN/m	q_theor_, kN/m	q_exp_, kN/m	(q_pl_/q_theor_) × 100%	(q_pl_/q_exp_) × 100%	(q_theor_/q_exp_) × 100%
S01	3.056	2.887	4.172	105.9	73.3	69.2
S02	3.056	3.511	4.053	87.0	75.4	86.6
S03	4.227	5.32	6.34	79.5	66.7	83.9
S04	1.72	1.805	2.525	95.3	68.1	71.5
S05	1.839	2.191	2.88	83.9	63.9	76.1
S06	2.406	3.309	4.127	72.7	58.3	80.2
S07	2.796	3.06	3.509	91.4	79.7	87.2
S08	2.915	3.06	3.462	95.3	84.2	88.4
S09	2.915	3.06	3.391	95.3	86.0	90.2
S10	1.299	1.797	2.177	72.3	59.7	82.5
S11	2.118	2.709	3.294	78.2	64.3	82.2
S12	1.811	2.261	2.599	80.1	69.7	87.0

## Data Availability

The original contributions presented in this study are included in the article. Further inquiries can be directed to the corresponding author.
